# The Metabolic Signature of AML Cells Treated With Homoharringtonine

**DOI:** 10.3389/fonc.2022.931527

**Published:** 2022-06-14

**Authors:** Yulong Zhang, Na Li, Zhiguang Chang, Huabin Wang, Hanzhong Pei, Dengyang Zhang, Qi Zhang, Junbin Huang, Yao Guo, Yuming Zhao, Yihang Pan, Chun Chen, Yun Chen

**Affiliations:** ^1^ Edmond H. Fischer Translational Medical Research Laboratory, Scientific Research Center, The Seventh Affiliated Hospital, Sun Yat-Sen University, Shenzhen, China; ^2^ Department of Pediatrics, The Seventh Affiliated Hospital of Sun Yat-Sen University, Shenzhen, China

**Keywords:** homoharringtonine, AML, ether lipid metabolism, RNA-seq, choline metabolism

## Abstract

Acute myeloid leukemia (AML) is a hematologic malignancy. The overall prognosis is poor and therapeutic strategies still need to be improved. Studies have found that abnormalities in metabolisms promote the survival of AML cells. In recent years, an increasing number of studies have reported the effectiveness of a protein synthesis inhibitor, homoharringtonine (HHT), for the treatment of AML. In this study, we demonstrated that HHT effectively inhibited AML cells, especially MV4-11, a cell line representing human AML carrying the poor prognostic marker FLT3-ITD. We analyzed the transcriptome of MV4-11 cells treated with HHT, and identified the affected metabolic pathways including the choline metabolism process. In addition, we generated a line of MV4-11 cells that were resistant to HHT. The transcriptome analysis showed that the resistant mechanism was closely related to the ether lipid metabolism pathway. The key genes involved in these processes were *AL162417.1*, *PLA2G2D*, and *LPCAT2* by multiple intergroup comparison and Venn analysis. In conclusion, we found that the treatment of HHT significantly changed metabolic signatures of AML cells, which may contribute to the precise clinical use of HHT and the development of novel strategies to treat HHT-resistant AML.

## Introduction

Acute myeloid leukemia (AML) is a heterogeneous hematologic malignancy featured by a proliferation of myeloblasts that are unable to undergo normal differentiation (1). It is the most prevalent form of leukemia in adults and the second major type of acute leukemia in children (2, 3). The disease progresses rapidly, with a survival of only a few weeks to a few months if untreated. In the United States, the 5-year overall survival rate for adults with relapsed/refractory AML (excluding APL) is approximately 10% (4).

Homoharringtonine (HHT) is a plant alkaloid that inhibits protein synthesis and has anti-tumor properties. In October 2012, HHT was approval by the U.S. Food and Drug Administration (FDA) for the treatment of chronic or accelerated CML after failure treatment of 2 or more tyrosine kinase inhibitors (5, 6). HHT has been used in China for more than 40 years to treat AML (7). A multicenter phase 3 trial found that the HAA (homoharringtonine-cytarabine-aclarubicin) regimen could be used as an alternative induction therapy for untreated AML, particularly in those patients with favorable and intermediate cytogenetics (8). Previous studies have found multiple mechanisms of HHT in treating AML, including targeting FLT3 pathway, antioxidant defense, SP1/TET1/5hmC pathway, TRAIL and p53 pathway, NF-κB pathway and the expression of myosin-9 and eIF4E (9–15). The mechanisms of resistance to HHT in AML include the reactivation of PI3K/AKT signaling pathway and the overexpression of MDR1, MRP1, P170-glycoprotein, MCL-1, and MAP4K1 (16–20). In addition, an increasing number of studies have reported synergistic effects of HHT combined with FLT3 inhibitors for the treatment of AML with FLT3-ITD (9, 21, 22). Recent research has shown that the treatment of FLT3 inhibitor gilteritinib leads to reprogramming of glycolipid metabolism in the tumor microenvironment, resulting in early and late drug resistance (23). Also, HHT in combination with glutaminase inhibitor CB-839 exacerbates AML mitochondrial reactive oxygen species and apoptosis (24). Venetoclax is a potent and selective oral inhibitor of BCL-2, a key regulator of mitochondrial energy metabolism that plays an important role in the survival of AML stem cells (25, 26). A study has shown a strong synergistic effect of venetoclax and HHT in diffuse large B-cell lymphoma *in vitro* and *in vivo* (27). Although a major progress has been made, the detailed mechanisms of effectiveness and resistance in HHT-based therapy in AML still need to be explored. And, the mechanisms of HHT metabolism-related studies in AML have not been reported.

Deregulating cellular metabolism is a hallmark of cancer (28). In AML, knockdown of ANRIL leads to decreased glucose uptake and inhibits survival of leukemia cells (29). Inhibition of glycolysis attenuates the proliferation of AML cells and enhances the cytotoxicity of Ara-C (30). Also, acetyl-CoA carboxylase 1(ACC1) was found to be the rate-limiting enzyme for fatty acid synthesis, and upregulation of ACC1 protein expression has the potential to be an effective strategy for the treatment of AML (31). Therefore, metabolic abnormalities in AML are highly involved in disease progression and resistance to the treatment, which need to be further investigated. In this study, we explored the change in transcriptome of HHT-treated AML cells and identified key metabolism pathways that may affect sensitivity and resistance of AML cells to HHT.

## Materials and Methods

### Cell Culture

Human AML cell lines MOLM-13 and MV4-11 were obtained from DSMZ (Braunschweig, Germany) and ATCC (VA, USA). The cells were maintained in RPMI 1640 (Hyclone, Thermo Fisher Scientific, USA) supplemented with 10% fetal bovine serum (FBS) (Gemini, USA), 100 U/ml penicillin, and 100µg/ml streptomycin (Hyclone, Thermo Fisher Scientific, USA) at 37°C in a humidified atmosphere containing 5% CO_2_.

### Cell Viability Assay and Flow Cytometry

For the cell viability assay, 6 × 10^4^ cells were plated in each well in a 96-well plate with complete RPMI 1640 supplemented with 10% FBS. The plate was incubated for 24 hours before detection. Cell viability was measured by the cell counting kit-8 (CCK-8, Solaribio, Beijing, China). For the apoptotic assay, cells were seeded in 24-well plates at 0.75× 10^6^ cells/well and incubated for 18 hours and subjected to flow cytometry. The Annexin V-FITC Apoptosis Detection Kit was obtained from DOJINDO (Japan) (AD10-50). Flow cytometric analysis was performed by a Cytoflex flow cytometer (Beckman, USA).

### Transcriptome Analysis

We obtained the resistant MV4-11 strain (R) by gradually increasing the concentration of HHT and kept the cells in culture with 10 nM for about 10 months. After obtaining the resistant strain, we cultured the resistant strain off-drug for about 3 months to obtain the detoxified strain (O). Together with the parental MV4-11 cells (N), R and O cells were cultured with or without 10 nM HHT for 6 hours. The samples were designated N0 and N6 for parent cells without or with HHT, O0 and O6 for off-drug cells without or with HHT, and R0 and R6 for resistant R cells with or without HHT, respectively. Cells were collected in lyophilization tubes and frozen in liquid nitrogen for 10 minutes. The isolation of RNA and next-generation of sequencing were performed by Genedenovo Biotechnology Co., Ltd (Guangzhou, China). Raw data and normalized gene expression data are deposited in the sequence read archive database under accession numbers PRJNA832421. Gene Ontology (GO) and Kyoto Encyclopedia of Genes and Genomes (KEGG) pathways were performed by Omicshare tools (https://www.omicshare.com/tools/). Gene Set Enrichment Analysis (GESA) was performed by using software GSEA and MSigDB (32).

### Statistics

Data visualizing and statistical analysis were performed using GraphPad Prism 8.0 (GraphPad Software Inc., CA, USA). Differences between experimental groups were analyzed using unpaired Student t test. *p* value < 0.05 was considered significant.

## Results

### HHT Significantly Induced Apoptosis of MV4-11 and MOLM13

**Figure 1:**
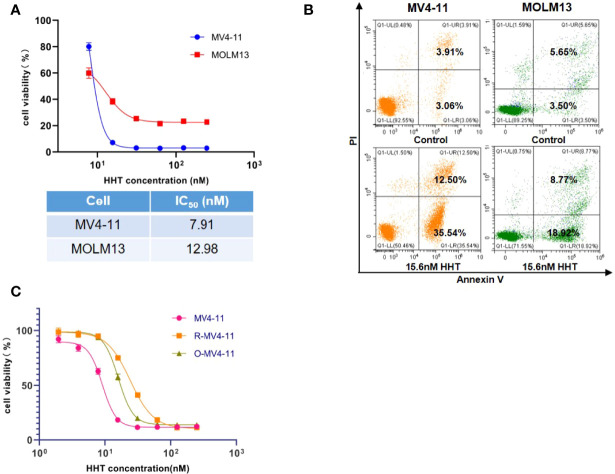
AML cell lines were sensitive to HHT and the establishment of HHT-resistant cells. MV4-11, MOLM13 cells were treated with different concentrations of HHT for 24 hours, and cell viability was detected by CCK-8 **(A)**. AML cells were treated without or with 15.6 nM HHT for 18 hours, and analyzed by flow cytometry **(B)**. Cells were treated with different concentrations of HHT for 24 hours. The cell viability of MV4-11, MV4-11 resistant strain (R-MV4-11), and off-drug strain (O-MV4-11) was detected by CCK-8 **(C)**.

In the cell viability assay, we found that MV4-11 and MOLM13 were sensitive to the treatment of HHT, with IC_50_ of 7.92 nM and 12.98 nM, respectively (**Figure 1A**). Flow cytometry showed that HHT induced significant apoptosis in MV4-11 and MOLM13 (**Figure 1B**). To explore the resistant mechanism in HHT-treated AML cells, we established MV4-11 that resisted to HHT by gradually increasing the concentration of HHT in the medium. The cell viability assay showed that MV4-11 resistant strain and off-drug strain were less sensitive to the treatment of HHT, compared with the original MV4-11 (**Figure 1C**).

### Choline Metabolism Was Associated With HHT Treatment in AML

**Figure 2:**
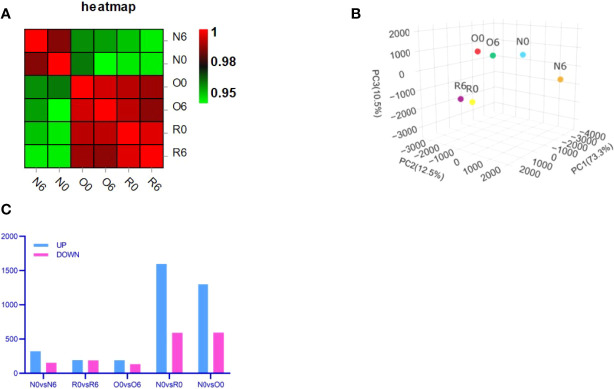
Transcriptome analysis showed an ideal cell line modeling. Correlation heat map **(A)** and Principal Component Analysis (PCA) **(B)** were used to assess intra- and inter-group differences in the samples. Bar charts showed the number of significantly different genes between groups (FDR < 0.05, multiple of difference greater than or equal to 2) **(C)**.

We performed transcriptome analysis in 3 strains of MV4-11 cells treated by HHT or out of HHT, including the original strain (N0 and N6), the HHT-resistant strain (R0 and R6), and the off-drug strain (O0 and O6). The genetic heat map analysis and principal component analysis revealed a large inter-group variability and a small intra-group variability (**Figures 2A, B**), which was in accordance with the number of up- and down-regulated genes in each group (**Figure 2C**). These data indicate an ideal cell line modeling in our transcriptome analysis.

**Figure 3:**
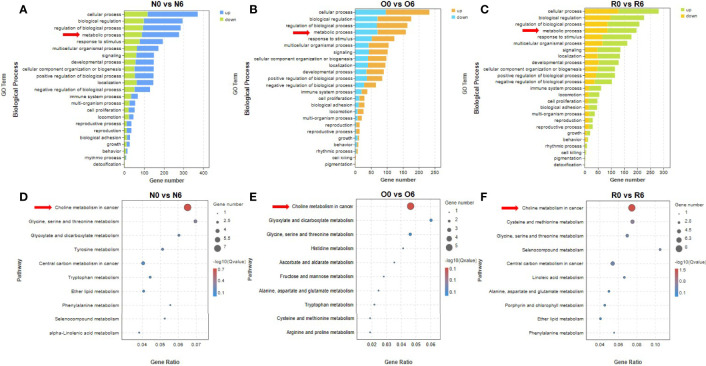
The drug efficacy mechanism of HHT was related to choline metabolism. Biological processes significantly affected in different cell lines with 10 nM HHT treatment for 6 hours **(A, B)** or HHT withdraw for 6 hours in the resistant line were found by GO enrichment analysis **(C)**. HHT was found to significantly affect metabolism-related signaling pathways by KEGG enrichment analysis **(D–F)**.

**Figure 4:**
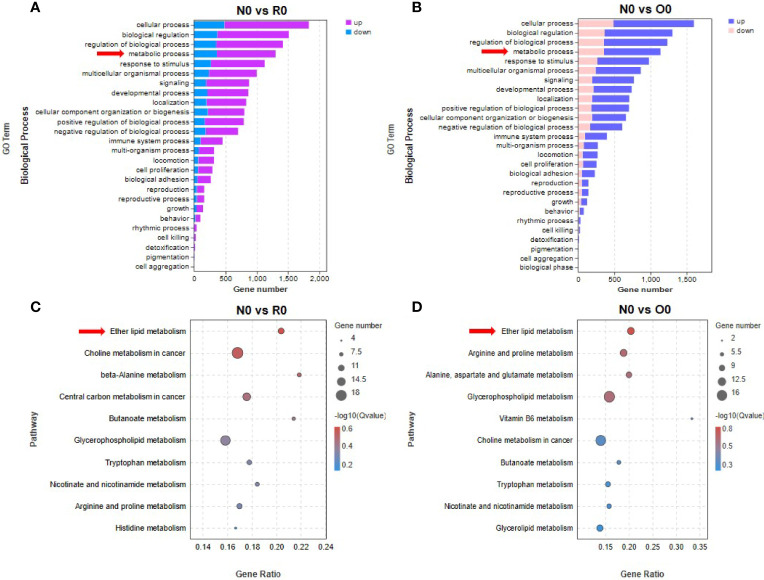
Ether lipid metabolism was associated with the mechanism of HHT resistance. By GO enrichment analysis, several biological processes were found to be significantly affected in long-term HHT-treated cell lines **(A, B)**. Significant effects of HHT on metabolism-related signaling pathways were found by KEGG enrichment analysis **(C, D)**.

In groups of N0 vs N6, O0 vs O6, and R0 vs R6, genes in metabolism processes were found to be significantly enriched by GO enrichment analysis (**Figures 3A–C**). To clarify the specific metabolic pathways, we performed KEGG enrichment analysis and found that genes in the choline metabolism pathway were significantly enriched after short-term intervention with HHT or out of HHT (N0 vs N6, *p* = 0.01; O0 vs O6, *p* = 0.02; R0 vs R6, *p* = 0.001) (**Figures 3D–F**). In contrast, this metabolic pathway was not significantly enriched in groups of long-term intervention of HHT (**Figures 4C, D**). Therefore, we speculated that the choline metabolism pathway is involved in the anti-leukemia effect of HHT in AML.

### Ether Lipid Metabolism Was Associated With the Resistance to HHT in AML

In groups of N0 vs R0 and N0 vs O0, GO enrichment analysis revealed that genes in metabolism-related biological processes were significantly enriched (**Figures 4A, B**). To clarify specific metabolic pathways, we performed KEGG enrichment analysis and found that the genes in ether lipid metabolism pathway were significantly enriched after long-term HHT intervention (N0 vs R0, *p*= 0.048) (**Figure 4C**). In the off-drug group, genes in this pathway were also significantly enriched (N0 vs O0, *p* = 0.02) (**Figure 4D**). In contrast, this metabolic pathway was not significantly enriched in HHT short-term intervention groups (N0 vs N6, *p* = 0.30; O0 vs O6, *p* = 0.51; R0 vs R6, *p* = 0.25). Therefore, we conclude that the ether lipid metabolism pathway is involved in promoting the development of resistance to HHT in AML cells. Interestingly, in the N0 vs O0 group, “arginine and proline metabolism” (*p* = 0.037), “alanine, aspartate and glutamate metabolism” (*p* = 0.044), and “glycerophospholipid metabolism” (*p* = 0.045) were also significantly enriched, and these pathways may contribute to the re-sensitization of O0 strain to HHT.

### AL162417.1, PLA2G2D, and LPCAT2 Were Key Genes in the Metabolic Signature of HHT-Treated AML Cells

**Figure 5:**
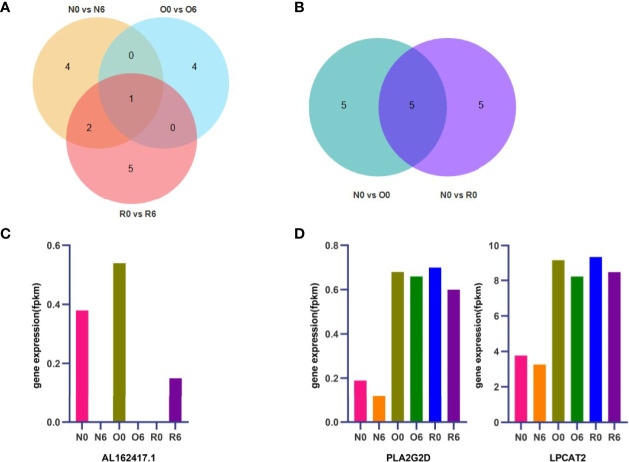
*AL162417.1, PLA2G2D*, and *LPCAT2* are candidate genes for drug efficacy and resistance. The core genes of drug efficacy and resistance related pathways identified by KEGG enrichment analysis and the shared key genes were found by Venn diagram **(A, B)**. Expression of key genes for drug efficacy and resistance were detected by RNA-seq **(C, D)**.

By using KEGG enrichment analysis, we identified the core set of genes associated with the treatment of HHT in AML cells. Drug efficacy gene of HHT was *AL162417.1* (**Figures 5A, C**). The key genes of resistance to HHT were *PLA2G2D*, *LPCAT2*, *UGT8*, *CHPT1*, and *GDPD1* (**Figure 5B**).

Since the fluctuation of drug resistance genes should not be significant changed after short-term administration or withdrawal of HHT, *CHPT1*, *UGT8*, and *GDPD1* were excluded (**Figure S1A**). The key drug resistance-related genes were identified as *PLA2G2D* and *LPCAT2* (**Figure 5D**). In addition, we found that *PLA2G2D* and *LPCAT2* were significantly enriched in the group of N0 vs O0 by GSEA (**Figure S1B**), which further validated *PLA2G2D* and *LPCAT2* as key HHT-resistance genes in AML cells.

## Discussion

Tumorigenesis and progression require metabolic reprogramming of cancer cells (33, 34), and understanding this process is important for tumor diagnosis, treatment, and prognosis. Abnormalities in choline metabolism have been reported in a variety of tumors. Several studies have reported that C-choline PET/CT can detect biochemical recurrent prostate cancer recurrence and survival (35, 36). In Glioblastomas cell lines U87MG and GBM5, choline increases tumor cell proliferation and anti-apoptosis by activating α7- and α9-containing nicotinic receptors and promoting AKT and ERK phosphorylation (37). The increased choline uptake was reported in endometrial cancer cell lines compared to normal endometrial stromal cells (38). CHPT1 drives cytidine diphosphate-choline (CDP-Cho) to generate phosphatidylcholine (PtdCho). Knockdown of CHPT1 inhibits the growth and proliferation of breast cancer cells, and *in vivo* experiments have shown that knockdown of CHPT1 inhibits the early metastasis of breast cancer cells (39). Genomic pooling analysis of colorectal cancer gut microbes shows that the choline trimethylamine-lyase gene is overexpressed in colorectal cancer (40). It is reported that in some types of tumors, the upregulation of choline metabolic pathway may lead to proliferation and anti-apoptosis of tumor cells. Wang, Musharraf et al. found significant alterations in choline metabolism in AML patients (41, 42). Besides, increased phosphorylcholine (PCho) was found to be a common feature of all observed AML cell lines (43). However, the specific pathological significance of abnormal choline metabolism in AML has not been revealed. In this study, we found that the drug efficacy mechanism of HHT is closely related to the choline metabolism, and further identified key genes in the pathway. Note that *AL162417.1* is a novel protein-coding gene that is a homolog of *RALGDS*, which activates CHK (44), and promotes the production of PCho. Therefore, *AL162417.1* could serve as a potential target of HHT in treating AML cells. The current findings on this gene are limited, which deserves further in-depth studies.

Ether lipids play a role in membrane fusion, participate in cellular differentiation and signaling, and act as endogenous antioxidants (45). Ether lipid levels have been shown to elevate in tumors. The enzyme alkylglycer-onephosphate synthase (AGPS) is upregulated in several types of aggressive human cancer cells and primary tumors, and knockdown of AGPS in cancer cells decreases survival of tumor cells, cancer invasiveness, and tumor growth [24]. In this study, we found that HHT resistance mechanism is closely related to the ether lipid metabolism pathway, and further identified key genes as *PLA2G2D* and *LPCAT2*. *PLA2G2D* encodes a secreted member of the phospholipase A2 family, which produces lysophospholipids and free fatty acid and involves in ether lipid metabolism. Studies show that oxidative stress may induce the expression of *PLA2G2D* in mouse and human monocyte-derived macrophages through lipid peroxidation. The high expression of *PLA2G2D* increases viral infection, and mice lacking *PLAG2D* are protected from COVID-19 invasion (46, 47). Besides, it has been reported that upregulation of *PLA2G2D* could be a potential biomarker for cancer immunotherapy (48). *LPCAT2* is involved in ether lipid metabolism and is a member of the family of enzymes encoding lysophospholipid acyltransferases. The expression level of *LPCAT2* is positively correlated with aggressive prostate cancer (49). Importantly, *LPCAT2* mediated lipid droplet production has been shown to promote resistance to chemotherapy in colorectal cancer (50). Therefore, *LPCAT2* is more promising candidate genes for HHT resistance. Furthermore, by ether lipids metabolism KEGG signaling pathway diagram (**Figure S2**), we found that PLA2G2D and LPCAT2 are involved in the conversion between lysoPAF and plasmanylcholine, but overall promote lysoPAF to plasmanylcholine production.

In summary, our study found that the mechanism of HHT efficacy in AML is related to choline metabolism, and the key candidate gene was *AL162417.1*. The resistance mechanism is related to ether lipid metabolism, and the core resistance candidate genes are *PLA2G2D* and *LPCAT2*. This study provides a new perspective for the rational clinical use of HHT and the development of new HHT-resistant treatment strategies.

## Data Availability Statement

The datasets presented in this study can be found in online repositories. The names of the repository/repositories and accession number(s) can be found in the article/**Supplementary Material**.

## Author Contributions

YP, CC, and YC conceived the project. YLZ, NL, ZC and HW performed the experiments. QZ, DZ, JH, YG and YMZ analyzed data. YLZ, ZC, and HW wrote the manuscript. All authors contributed to the article and approved the submitted version.

## Funding

The work was supported by Guangdong Provincial Key Laboratory of Digestive Cancer Research (No. 2021B1212040006). We also thank Guangdong Basic and Applied Basic Research Foundation (2019A1515110121) to support the design of the study, National Natural Science Foundation of China (NSFC, Grant No. 82000150), Shenzhen Healthcare Research Project (Grant No. SZLY2018001), and Shenzhen Science and Technology Innovation Commission (JCYJ20190814164601648 and JCYJ20210324123003009) to support sample collection and analysis, and Sanming Project of Medicine in Shenzhen (No. SZSM202011004) to support the manuscript preparation and publication.

## Conflict of Interest

The authors declare that the research was conducted in the absence of any commercial or financial relationships that could be construed as a potential conflict of interest.

## Publisher’s Note

All claims expressed in this article are solely those of the authors and do not necessarily represent those of their affiliated organizations, or those of the publisher, the editors and the reviewers. Any product that may be evaluated in this article, or claim that may be made by its manufacturer, is not guaranteed or endorsed by the publisher.

## References

[1] Newell LF Cook RJ. Advances in Acute Myeloid Leukemia. BMJ (2021) 375:n2026. doi: 10.1136/bmj.n2026 34615640

[2] Burd A Levine RL Ruppert AS Mims AS Borate U Stein EM. Precision Medicine Treatment in Acute Myeloid Leukemia Using Prospective Genomic Profiling: Feasibility and Preliminary Efficacy of the Beat Aml Master Trial. Nat Med (2020) 26(12):1852–8. doi: 10.1038/s41591-020-1089-8 PMC8530434 33106665

[3] Carter JL Hege K Yang J Kalpage HA Su Y Edwards H. Targeting Multiple Signaling Pathways: The New Approach to Acute Myeloid Leukemia Therapy. Signal Transduct Target Ther (2020) 5(1):288. doi: 10.1038/s41392-020-00361-x 33335095 PMC7746731

[4] DeWolf S Tallman MS. How I Treat Relapsed or Refractory Aml. Blood (2020) 136(9):1023–32. doi: 10.1182/blood.2019001982 PMC7453152 32518943

[5] Cortes J Lipton JH Rea D Digumarti R Chuah C Nanda N. Phase 2 Study of Subcutaneous Omacetaxine Mepesuccinate After Tki Failure in Patients With Chronic-Phase Cml With T315i Mutation. Blood (2012) 120(13):2573–80. doi: 10.1182/blood-2012-03-415307 PMC4916583 22896000

[6] Lu S Wang J. Homoharringtonine and Omacetaxine for Myeloid Hematological Malignancies. J Hematol Oncol (2014) 7:2. doi: 10.1186/1756-8722-7-2 24387717 PMC3884015

[7] Harringtonine Research Collaborative Group. Preliminary Clinical Evaluation of Harringtonine Alkaloids in the Treatment of Acute Leukemia. Zhejiang Tumor Newslett (1976) (01):56–62.

[8] Jin J Wang JX Chen FF Wu DP Hu J Zhou JF. Homoharringtonine-Based Induction Regimens for Patients With De-Novo Acute Myeloid Leukaemia: A Multicentre, Open-Label, Randomised, Controlled Phase 3 Trial. Lancet Oncol (2013) 14(7):599–608. doi: 10.1016/S1470-2045(13)70152-9 23664707

[9] Lam SS Ho ES He BL Wong WW Cher CY Ng NK. Homoharringtonine (Omacetaxine Mepesuccinate) as an Adjunct for Flt3-Itd Acute Myeloid Leukemia. Sci Transl Med (2016) 8(359):359ra129. doi: 10.1126/scitranslmed.aaf3735 27708062

[10] Zhang JJ Geng HY Liu L Zhang H. Synergistic Cytotoxicity of Homoharringtonine and Etoposide in Acute Myeloid Leukemia Cells Involves Disrupted Antioxidant Defense. Cancer Manage Res (2019) 11:1023–32. doi: 10.2147/cmar.S187597 PMC6349074 30774430

[11] Li C Dong L Su R Bi Y Qing Y Deng X. Homoharringtonine Exhibits Potent Anti-Tumor Effect and Modulates DNA Epigenome in Acute Myeloid Leukemia by Targeting Sp1/Tet1/5hmc. Haematologica (2020) 105(1):148–60. doi: 10.3324/haematol.2018.208835 PMC6939512 30975912

[12] Cao H Cheng Y You L Qian J Qian W. Homoharringtonine and Saha Synergistically Enhance Apoptosis in Human Acute Myeloid Leukemia Cells Through Upregulation of Trail and Death Receptors. Mol Med Rep (2013) 7(6):1838–44. doi: 10.3892/mmr.2013.1440 23620163

[13] Tan M Zhang Q Yuan X Chen Y Wu Y. Synergistic Killing Effects of Homoharringtonine and Arsenic Trioxide on Acute Myeloid Leukemia Stem Cells and the Underlying Mechanisms. J Exp Clin Cancer Res (2019) 38(1):308. doi: 10.1186/s13046-019-1295-8 31307525 PMC6631946

[14] Zhang T Shen S Zhu Z Lu S Yin X Zheng J. Homoharringtonine Binds to and Increases Myosin-9 in Myeloid Leukaemia. Br J Pharmacol (2016) 173(1):212–21. doi: 10.1111/bph.13359 PMC4813388 26448459

[15] Chen XJ Zhang WN Chen B Xi WD Lu Y Huang JY. Homoharringtonine Deregulates Myc Transcriptional Expression by Directly Binding Nf-Kappab Repressing Factor. Proc Natl Acad Sci U.S.A. (2019) 116(6):2220–5. doi: 10.1073/pnas.1818539116 PMC6369765 30659143

[16] Wang L You LS Ni WM Ma QL Tong Y Mao LP. Beta-Catenin and Akt Are Promising Targets for Combination Therapy in Acute Myeloid Leukemia. Leuk Res (2013) 37(10):1329–40. doi: 10.1016/j.leukres.2013.06.023 23867056

[17] Zhang W Lu Y Zhen T Chen X Zhang M Liu P. Homoharringtonine Synergy With Oridonin in Treatment of T (8, 21) Acute Myeloid Leukemia. Front Med (2019) 13(3):388–97. doi: 10.1007/s11684-018-0624-1 30206768

[18] Zhou DC Ramond S Viguie F Faussat AM Zittoun R Marie JP. Sequential Emergence of Mrp- and Mdr1-Gene Over-Expression as Well as Mdr1-Gene Translocation in Homoharringtonine-Selected K562 Human Leukemia Cell Lines. Int J Cancer (1996) 65(3):365–71. doi: 10.1002/(SICI)1097-0215(19960126)65:3<365::AID-IJC15>3.0.CO;2-9 8575859

[19] Chen P Zhan W Wang B You P Jin Q Hou D. Homoharringtonine Potentiates the Antileukemic Activity of Arsenic Trioxide Against Acute Myeloid Leukemia Cells. Exp Cell Res (2019) 376(2):114–23. doi: 10.1016/j.yexcr.2019.02.008 30763586

[20] Russo D Michelutti A Melli C Damiani D Michieli MG Candoni A. Mdr-Related P170-Glycoprotein Modulates Cytotoxic Activity of Homoharringtonine. Leukemia (1995) 9(3):513–6. 7885049

[21] Wang F Huang J Guo T Zheng Y Zhang L Zhang D. Homoharringtonine Synergizes With Quizartinib in Flt3-Itd Acute Myeloid Leukemia by Targeting Flt3-Akt-C-Myc Pathway. Biochem Pharmacol (2021) 188:114538. doi: 10.1016/j.bcp.2021.114538 33831397

[22] Zhang C Lam SSY Leung GMK Tsui SP Yang N Ng NKL. Sorafenib and Omacetaxine Mepesuccinate as a Safe and Effective Treatment for Acute Myeloid Leukemia Carrying Internal Tandem Duplication of Fms-Like Tyrosine Kinase 3. Cancer (2020) 126(2):344–53. doi: 10.1002/cncr.32534 31580501

[23] Joshi SK Nechiporuk T Bottomly D Piehowski PD Reisz JA Pittsenbarger J. The Aml Microenvironment Catalyzes a Stepwise Evolution to Gilteritinib Resistance. Cancer Cell (2021) 39(7):999–1014.e8. doi: 10.1016/j.ccell.2021.06.003 34171263 PMC8686208

[24] Gregory MA Nemkov T Park HJ Zaberezhnyy V Gehrke S Adane B. Targeting Glutamine Metabolism and Redox State for Leukemia Therapy. Clin Cancer Res (2019) 25(13):4079–90. doi: 10.1158/1078-0432.CCR-18-3223 PMC6642698 30940653

[25] Short NJ Konopleva M Kadia TM Borthakur G Ravandi F DiNardo CD. Advances in the Treatment of Acute Myeloid Leukemia: New Drugs and New Challenges. Cancer Discovery (2020) 10(4):506–25. doi: 10.1158/2159-8290.CD-19-1011 32014868

[26] Lagadinou ED Sach A Callahan K Rossi RM Neering SJ Minhajuddin M. Bcl-2 Inhibition Targets Oxidative Phosphorylation and Selectively Eradicates Quiescent Human Leukemia Stem Cells. Cell Stem Cell (2013) 12(3):329–41. doi: 10.1016/j.stem.2012.12.013 PMC3595363 23333149

[27] Klanova M Andera L Brazina J Svadlenka J Benesova S Soukup J. Targeting of Bcl2 Family Proteins With Abt-199 and Homoharringtonine Reveals Bcl2- and Mcl1-Dependent Subgroups of Diffuse Large B-Cell Lymphoma. Clin Cancer Res (2016) 22(5):1138–49. doi: 10.1158/1078-0432.CCR-15-1191 26467384

[28] Hanahan D. Hallmarks of Cancer: New Dimensions. Cancer Discovery (2022) 12(1):31–46. doi: 10.1158/2159-8290.CD-21-1059 35022204

[29] Sun LY Li XJ Sun YM Huang W Fang K Han C. Lncrna Anril Regulates Aml Development Through Modulating the Glucose Metabolism Pathway of Adipor1/Ampk/Sirt1. Mol Cancer (2018) 17(1):127. doi: 10.1186/s12943-018-0879-9 30134922 PMC6106744

[30] Chen WL Wang JH Zhao AH Xu X Wang YH Chen TL. A Distinct Glucose Metabolism Signature of Acute Myeloid Leukemia With Prognostic Value. Blood (2014) 124(10):1645–54. doi: 10.1182/blood-2014-02-554204 PMC5726328 25006128

[31] Ito H Nakamae I Kato J Yoneda-Kato N. Stabilization of Fatty Acid Synthesis Enzyme Acetyl-Coa Carboxylase 1 Suppresses Acute Myeloid Leukemia Development. J Clin Invest (2021) 131(12):e141529. doi: 10.1172/jci141529 PMC8203453 34128473

[32] Subramanian A Tamayo P Mootha VK Mukherjee S Ebert BL Gillette MA. Gene Set Enrichment Analysis: A Knowledge-Based Approach for Interpreting Genome-Wide Expression Profiles. Proc Natl Acad Sci U.S.A. (2005) 102(43):15545–50. doi: 10.1073/pnas.0506580102 PMC1239896 16199517

[33] Martinez-Reyes I Chandel NS. Cancer Metabolism: Looking Forward. Nat Rev Cancer (2021) 21(10):669–80. doi: 10.1038/s41568-021-00378-6 34272515

[34] Pedroza-Torres A Romero-Cordoba SL Justo-Garrido M Salido-Guadarrama I Rodriguez-Bautista R Montano S. Micrornas in Tumor Cell Metabolism: Roles and Therapeutic Opportunities. Front Oncol (2019) 9:1404. doi: 10.3389/fonc.2019.01404 31921661 PMC6917641

[35] Michaud L Touijer KA Mauguen A Zelefsky MJ Morris MJ Lyashschenko SK. C-Choline Pet/Ct in Recurrent Prostate Cancer: Retrospective Analysis in a Large U.S. Patient Series. J Nucl Med (2020) 61(6):827–33. doi: 10.2967/jnumed.119.233098 PMC7262219 31862801

[36] Giovacchini G Guglielmo P Mapelli P Incerti E Gajate AMS Giovannini E. C-Choline Pet/Ct Predicts Survival in Prostate Cancer Patients With Psa < 1 Ng/Ml. Eur J Nucl Med Mol Imaging (2019) 46(4):921–9. doi: 10.1007/s00259-018-4253-3 30631911

[37] Pucci S Fasoli F Moretti M Benfante R Di Lascio S Viani P. Choline and Nicotine Increase Glioblastoma Cell Proliferation by Binding and Activating Alpha7- and Alpha9- Containing Nicotinic Receptors. Pharmacol Res (2021) 163:105336. doi: 10.1016/j.phrs.2020.105336 33276105

[38] Trousil S Lee P Pinato DJ Ellis JK Dina R Aboagye EO. Alterations of Choline Phospholipid Metabolism in Endometrial Cancer Are Caused by Choline Kinase Alpha Overexpression and a Hyperactivated Deacylation Pathway. Cancer Res (2014) 74(23):6867–77. doi: 10.1158/0008-5472.CAN-13-2409 25267063

[39] Jia M Andreassen T Jensen L Bathen TF Sinha I Gao H. Estrogen Receptor Alpha Promotes Breast Cancer by Reprogramming Choline Metabolism. Cancer Res (2016) 76(19):5634–46. doi: 10.1158/0008-5472.CAN-15-2910 27457520

[40] Thomas AM Manghi P Asnicar F Pasolli E Armanini F Zolfo M. Metagenomic Analysis of Colorectal Cancer Datasets Identifies Cross-Cohort Microbial Diagnostic Signatures and a Link With Choline Degradation. Nat Med (2019) 25(4):667–78. doi: 10.1038/s41591-019-0405-7 PMC9533319 30936548

[41] Wang Y Zhang L Chen WL Wang JH Li N Li JM. Rapid Diagnosis and Prognosis of *De Novo* Acute Myeloid Leukemia by Serum Metabonomic Analysis. J Proteome Res (2013) 12(10):4393–401. doi: 10.1021/pr400403p 23998518

[42] Musharraf SG Siddiqui AJ Shamsi T Choudhary MI Rahman AU. Serum Metabonomics of Acute Leukemia Using Nuclear Magnetic Resonance Spectroscopy. Sci Rep (2016) 6:30693. doi: 10.1038/srep30693 27480133 PMC4969755

[43] Lo Presti C Fauvelle F Mondet J Mossuz P. The Differential Activation of Metabolic Pathways in Leukemic Cells Depending on Their Genotype and Micro-Environmental Stress. Metabolomics (2020) 16(1):13. doi: 10.1007/s11306-020-1633-z 31925544

[44] Gallego-Ortega D Ramirez de Molina A Ramos MA Valdes-Mora F Barderas MG Sarmentero-Estrada J. Differential Role of Human Choline Kinase Alpha and Beta Enzymes in Lipid Metabolism: Implications in Cancer Onset and Treatment. PloS One (2009) 4(11):e7819. doi: 10.1371/journal.pone.0007819 19915674 PMC2773002

[45] Dean JM Lodhi IJ. Structural and Functional Roles of Ether Lipids. Protein Cell (2018) 9(2):196–206. doi: 10.1007/s13238-017-0423-5 28523433 PMC5818364

[46] Vijay R Hua X Meyerholz DK Miki Y Yamamoto K Gelb M. Critical Role of Phospholipase A2 Group Iid in Age-Related Susceptibility to Severe Acute Respiratory Syndrome-Cov Infection. J Exp Med (2015) 212(11):1851–68. doi: 10.1084/jem.20150632 PMC4612096 26392224

[47] Wong LR Zheng J Wilhelmsen K Li K Ortiz ME Schnicker NJ. Eicosanoid Signaling Blockade Protects Middle-Aged Mice From Severe Covid-19. Nature (2022) 605(7908):146–51. doi: 10.1038/s41586-022-04630-3 PMC9783543 35314834

[48] Liu H Xu R Gao C Zhu T Liu L Yang Y. Metabolic Molecule Pla2g2d Is a Potential Prognostic Biomarker Correlating With Immune Cell Infiltration and the Expression of Immune Checkpoint Genes in Cervical Squamous Cell Carcinoma. Front Oncol (2021) 11:755668. doi: 10.3389/fonc.2021.755668 34733790 PMC8558485

[49] Williams KA Lee M Hu Y Andreas J Patel SJ Zhang S. A Systems Genetics Approach Identifies Cxcl14, Itgax, and Lpcat2 as Novel Aggressive Prostate Cancer Susceptibility Genes. PloS Genet (2014) 10(11):e1004809. doi: 10.1371/journal.pgen.1004809 25411967 PMC4238980

[50] Cotte AK Aires V Fredon M Limagne E Derangere V Thibaudin M. Lysophosphatidylcholine Acyltransferase 2-Mediated Lipid Droplet Production Supports Colorectal Cancer Chemoresistance. Nat Commun (2018) 9(1):322. doi: 10.1038/s41467-017-02732-5 29358673 PMC5778070

