# Supplementary material for: The Metabolic Signature of AML Cells Treated With Homoharringtonine
Source: Front Oncol. 2022 Jun 14;12:931527. doi: 10.3389/fonc.2022.931527 (PMC9237253; doi:10.3389/fonc.2022.931527)
Supplement: Supplementary file 1 [file DataSheet_1.docx]

Supplementary Material

**The metabolic signature of AML cells treated with homoharringtonine**

**Yulong Zhang ^1†^, Na Li^1†^, Zhiguang Chang^1†^, Huabin Wang^2†^, Hanzhong Pei^1^, Dengyang Zhang^1^, Qi Zhang^1^, Junbin Huang^2^, Yao Guo^1^, Yuming Zhao^1^, Yihang Pan^1*^, Chun Chen^2*^, Yun Chen^1*^**

^1^ Edmond H. Fischer Translational Medical Research Laboratory, Scientific Research Center, The Seventh Affiliated Hospital, Sun Yat-Sen University, Shenzhen, China, ^2^ Department of Pediatrics, The Seventh Affiliated Hospital of Sun Yat-Sen University, Shenzhen, China

*** Correspondence:**Dr. Yun Chen, Edmond H. Fischer Translational Medical Research Laboratory, Scientific Research Center, The Seventh Affiliated Hospital, Sun Yat-sen University, Shenzhen, 518107 Guangdong, China; phone: (0755)81207021; email: cheny653@mail.sysu.edu.cn

Dr. Chun Chen, Department of Pediatrics, The Seventh Affiliated Hospital, Sun Yat-sen University, Shenzhen, 518107, Guangdong, China. phone: (0755)81206752; email: chenchun@mail.sysu.edu.cn

Dr. Yihang Pan, Edmond H. Fischer Translational Medical Research Laboratory, Scientific Research Center, The Seventh Affiliated Hospital, Sun Yat-sen University, Shenzhen, 518107 Guangdong, China; phone: (0755)81207031; email: [panyih@mail.sysu.edu.cn](mailto:panyih@mail.sysu.edu.cn)

Figure S1


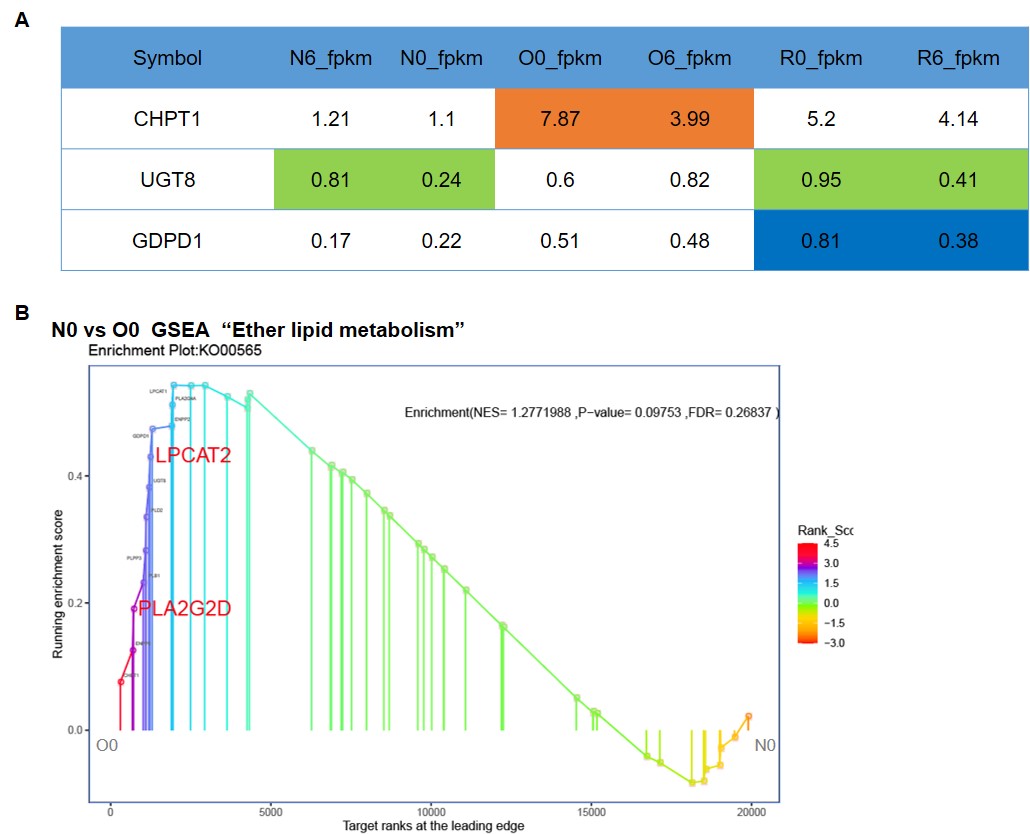


Figure S2


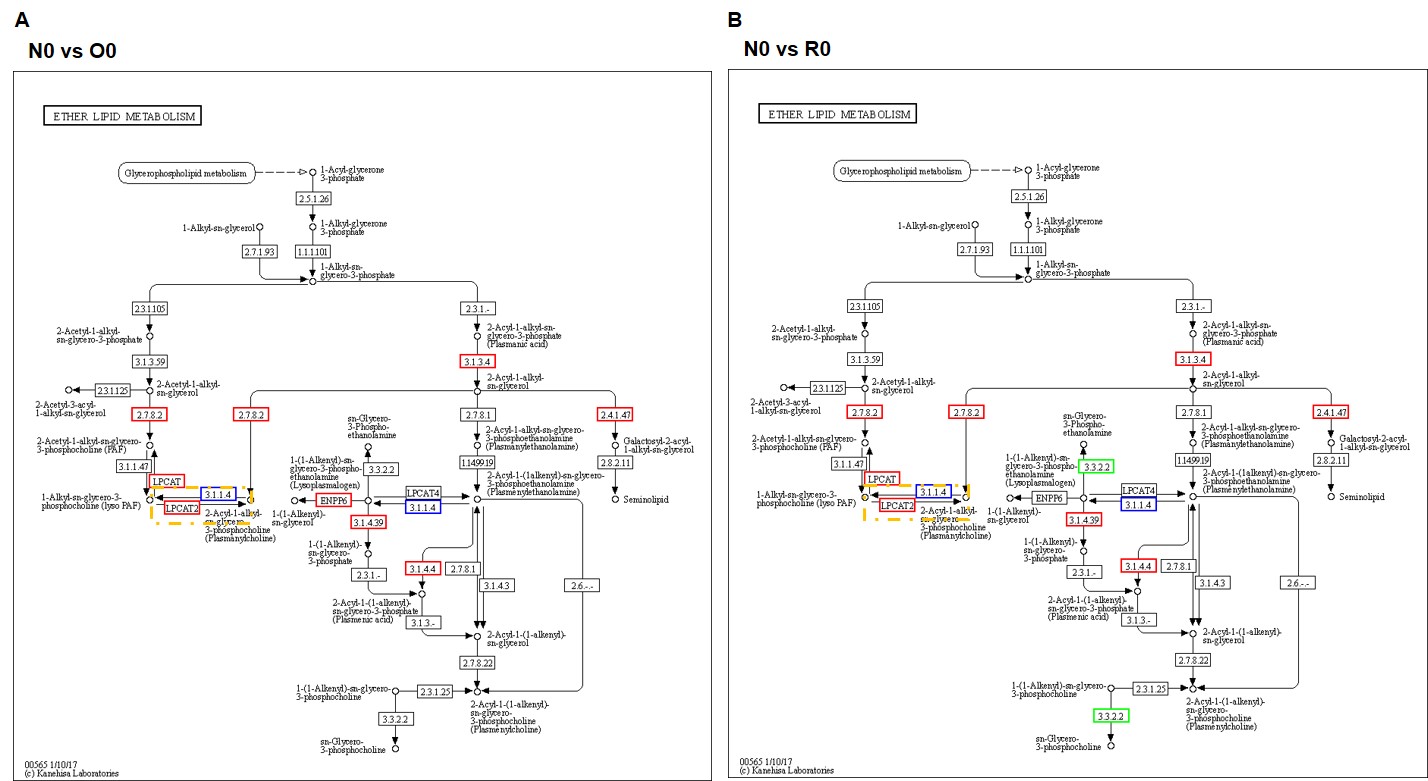


**Supplementary figure legends**

**Figure S1** Screening for drug-resistant genes. Drug resistance-related genes were identified by Venn analysis **(A)**. GSEA of the “Ether lipid metabolism” signaling pathway was in N0 vs O0 **(B)**.

**Figure S2** KEGG enrichment analysis. The “Ether lipid metabolism” signaling pathway was in N0 vs O0 (**A**) and N0 vs R0 (**B**). 3.1.1.4 is a gene set that includes *PLA2G2D*.
